# Infection risk in cable cars and other enclosed spaces

**DOI:** 10.1111/ina.13094

**Published:** 2022-08-21

**Authors:** Ivan Lunati, Claudio Mucignat

**Affiliations:** ^1^ Laboratory of Multiscale Studies in Building Physics, Empa Dübendorf Switzerland

**Keywords:** air exchange rate, airborne transmission, COVID‐19, indoor risk, probability of infection, stochastic infection model, velocimetry

## Abstract

As virus‐laden aerosols can accumulate and remain suspended for hours in insufficiently ventilated enclosed spaces, indoor environments can heavily contribute to the spreading of airborne infections. In the COVID‐19 pandemics, the role possibly played by cable cars has attracted media attention following several outbreaks in ski resort. To assess the real risk of infection, we experimentally characterize the natural ventilation in cable cars and develop a general stochastic model of infection in an arbitrary indoor space that accounts for the epidemiological situation, the virological parameters, and the indoor characteristics (ventilation rate and occupant number density). As a results of the high air exchange rate (we measured up to 180 air changes per hour) and the relatively short duration of the journey, the infection probability in cable cars traveling with open windows is remarkably lower than in other enclosed spaces such as aircraft cabins, train cars, offices, classrooms, and dining rooms. Accounting for the typical duration of the stay, the probability of infection during a cable‐car ride is lower by two to three orders of magnitude than in the other examples considered (the highest risk being estimated in case of a private gathering in a poorly ventilated room). For most practical purposes, the infection probability can be approximated by the inhaled viral dose, which provides an upper bound and allows a simple comparison between different indoor situations once the air exchange rate and the occupant number density are known. Our approach and findings are applicable to any indoor space in which the viral transmission is predominately airborne and the air is well mixed.

**Practical Implications**

By means of field velocimetry, we characterized the natural ventilation in cable cars and measured between 40 and 180 air changes per hour when windows are open. We devised a stochastic model of infection indoors and provided a simple approximated formula to calculate the risk of infections in practice. Traveling with open windows reduces the risk of infection by at least one order of magnitude. Due to the high air exchange rate and the short duration of the stay, the risk of infection in cable cars is much lower than in other means of transportation or in building spaces.

## 1 INTRODUCTION

The COVID‐19 pandemic (caused by the virus named SARS‐CoV‐2) has dramatically exemplified the disrupting potential of viruses transported through the air, which can rapidly and ubiquitously spread among a vast portion of the population leading to tragic medical, social, and economic consequences. Virus‐laden droplets and aerosols are emitted by infectious persons during respiratory events (such as breathing, speaking, coughing, and sneezing) and can infect a susceptible individual if the intake of viral copies becomes sufficiently large. Based on the classic analysis by Wells,^1^ the transmission through respiratory particles is traditionally classified into droplet route and airborne route. In the droplet route, the conjunctiva or the buccal and nasal mucosae of a susceptible individual are infected by large respiratory droplets that are emitted by a symptomatic individual which would otherwise rapidly settle by gravity; in contrasts, the airborne route is characterized by the presence of infectious viral copies in very small droplets and aerosols that can accumulate in enclosed spaces and remain in the air for hours. This classification often relies on rather arbitrary cutoff sizes (e.g., about 5 μm in Ref. [2]), but the involved physical processes are much more complex as intermediate droplets can evaporate into aerosols depending on ambient conditions (see, e.g., Refs [3, 4, 5, 6]).

Although at the very beginning of the pandemic spreading of the infection through surfaces (the fomite route) and the droplet route were considered the primary mechanisms of infection (see, e.g., Refs [2, 7, 8]), attention has been immediately drawn to the importance of the airborne route.^9^, ^10^ With the time, the role of surfaces has been reconsidered and scaled back^11^, ^12^ and there has been mounting evidence that airborne transmission plays a significant role in the COVID‐19 pandemic (see, e.g., Refs [3, 13, 14, 15]). This awareness about airborne transmission has led to increase the attention paid to enclosed spaces, where potentially virus‐laden aerosol can accumulate and remain suspended in the air for hours, leading to an increased risk of infection if ventilation is poor.^16^

Indeed, many studies report higher probability of infection indoors. For instance, Nissen et al.^17^ performed an epidemiological analysis hinting at airborne infection in a hospital ward. Kasper et al.^18^ reported an outbreak on an aircraft carrier and showed that the crew members working in confined areas (such as the engine room) were exposed to a higher risk of infection. By means of epidemiological data, measurements, and computational fluid dynamics, Li et al.^19^ suggested that poor indoor‐air management can potentially lead to airborne transmission in restaurants. Aerosol transmission of SARS‐CoV‐2 has also been reported in superspreading events^20^ and in poorly ventilated courtrooms.^21^

Public transport has also been indicated as possibly playing a major role in the spreading of COVID‐19 disease. Despite the comparatively shorter stay of the passengers, train, busses, and aircraft are characterized by high passenger density and turnover. This is the case also for cable cars and cableway gondolas, that have received major attention by mass media at the beginning of the pandemic, leading to the shutdown of ski‐resort operation in most countries with severe economic consequences.^22^ This was fueled by several outbreaks that have been observed in European ski resorts, particularly in Austria^23^, ^24^ and Switzerland.^25^ Recently, Gianfredi et al.^22^ reviewed several studies about outbreaks in ski resort and suggested that most infection clusters could be ascribed at public gathering rather than to recreational skiing. Nevertheless, the debate about the role of cable cars has continued, partly because the large number of passengers and perceived passenger density, and partly because of the lack of quantitative knowledge about natural ventilation in cable cars and cableway gondolas.

Our objectives are to experimentally characterize the natural ventilation in cable cars, to assess the probability of airborne transmission, and to compare it with airborne transmission in other enclosed spaces such as aircraft cabins, train cars, offices, classrooms, and dining rooms. To this end, we develop a general stochastic infection model that describes indoor airborne transmission through viral aerosols. The resulting infection probability and the individual risk of infection are functions of the epidemiological situation, the virological parameters, and the characteristics of the indoor space, such as ventilation rate and passenger number density.

## 2 THE PROBABILITY OF INFECTION IN INDOOR SPACES

### 2.1 The quantum of infection

The probability of infection of a susceptible person depends on the cumulative viral intake. As many parameters remain insufficiently known and the exposure dose causing infection varies across individuals, the problem is better addressed in a probabilistic framework. In case of an airborne disease, the exposure dose depends on the viral concentration of the inhaled air, q [m${}^{-3}$], and the number of new infections can be described by writing the evolution of the number of a susceptible individuals, S [−], as a law of mass action,

(1) $$\frac{d}{\mathrm{dt}}S\left(t\right)=-\mathrm{bq}\left(t\right)S\left(t\right),$$

where the proportionality constant b [m${}^{3}$/s] accounts for the probability of infection and is proportional to the air intake rate. In epidemiology, the law of mass action is widely used for environmentally mediated diseases (see, e.g., Refs [26, 27, 28]) and is also at the basis of compartmental models (see, e.g., Refs [29, 30, 31, 32]). Integrating Equation 1, we obtain the probability of a new infection,

(2) $$P_{\inf}\left(t\right)=1-\frac{S\left(t\right)}{S_{0}}=1-e^{-Q\left(t\right)},$$

where $S_{0}=S\left(0\right)$ is the number of susceptible individuals at time zero, and we have defined,

(3) $$Q\left(t\right)=\int_{0}^{t}\mathrm{bq}\left(t^{\prime }\right)dt^{\prime }.$$

If we assume that b is simply the breathing rate (or pulmonary rate) of the susceptible individual, then $Q\left(t\right)$ [−] is the infectious dose inhaled by a susceptible individual over a period of time t. When a susceptible individual has inhaled a quantity of viral copies corresponding to Q=1, the probability of infection is $P_{\inf}=1-e^{-1}$; this amount of viral copies is called *quantum of infection* ^33^ and is used as the measurement unit of the infectious dose. In other words, by assuming that b is simply the breathing rate, the probability of infection is taken into account by measuring the amount of viral copies in an appropriate unit, called the *quantum of infection*. Consistently, the viral concentration in the inhaled air, q, is measured of quanta of viral copies per cubic meter [m${}^{-3}$]. Notice that the equations above remain valid also if the breathing rate is a function of time: the infection probability depends the integral quanta of infection inhaled by the susceptible individual, regardless of the origin of the temporal variations.

### 2.2 The viral concentration in the air

The viral concentration in an enclosed space depends on the balance between the emission of viral copies by infectious individuals and their removal by ventilation, deposition, inactivation, or inhalation. Assuming well‐mixed conditions, the balance equation for the viral concentration in the indoor air can be written as

(4) $$V\frac{d}{\mathrm{dt}}q\left(t\right)=\theta_{q}-q\left(t\right)\left(E+\mathrm{\lambda V}+B\right),$$

where V is the indoor volume; $\theta_{q}$ is the quanta emission rate by the infectious individuals (i.e., the emission rate of viral copies measured in quanta of infection) [1/s]; E [m${}^{3}$/s] is the ventilation rate replacing indoor air by outdoor fresh air (assumed to have zero viral concentration); λ [1/s] describes the decrease of the infectious viral copies present in the air due to deposition or inactivation (e.g., by ultraviolet radiation or disinfection); and B is the total respiratory rate of all individuals in the enclosed space, and describes virus removal by inhalation and subsequent deposition in their respiratory systems (notice that this term can dominate the deposition rate in case of relatively large occupant‐number density and is a non‐negligible removal process in absence of ventilation). Defining the air exchange rate,

(5) $$\tau_{e}^{-1}=\frac{E}{V},$$

(where $\tau_{e}$ [s] is the air exchange time) and the virus removal rate,

(6) $$\tau_{q}^{-1}=\frac{E+\mathrm{\lambda V}+B}{V}=\tau_{e}^{-1}\left(1+\mathrm{\lambda V}/E+B/E\right),$$

(where $\tau_{q}$ [s] is the virus removal time) we can write the evolution of the viral concentration of the indoor air,

(7) $$\frac{d}{\mathrm{dt}}q\left(t\right)=V^{-1}\theta_{q}-\tau_{q}^{-1}q\left(t\right).$$

Integrating Equation 7 and assuming that the initial viral concentration is zero, we obtain the viral concentration as a function of time,

(8) $$q\left(t\right)=\frac{\tau_{q}\theta_{q}}{V}\left(1-e^{-t/\tau_{q}}\right).$$

### 2.3 The G‐N model and the probability of infection

**FIGURE 1:**
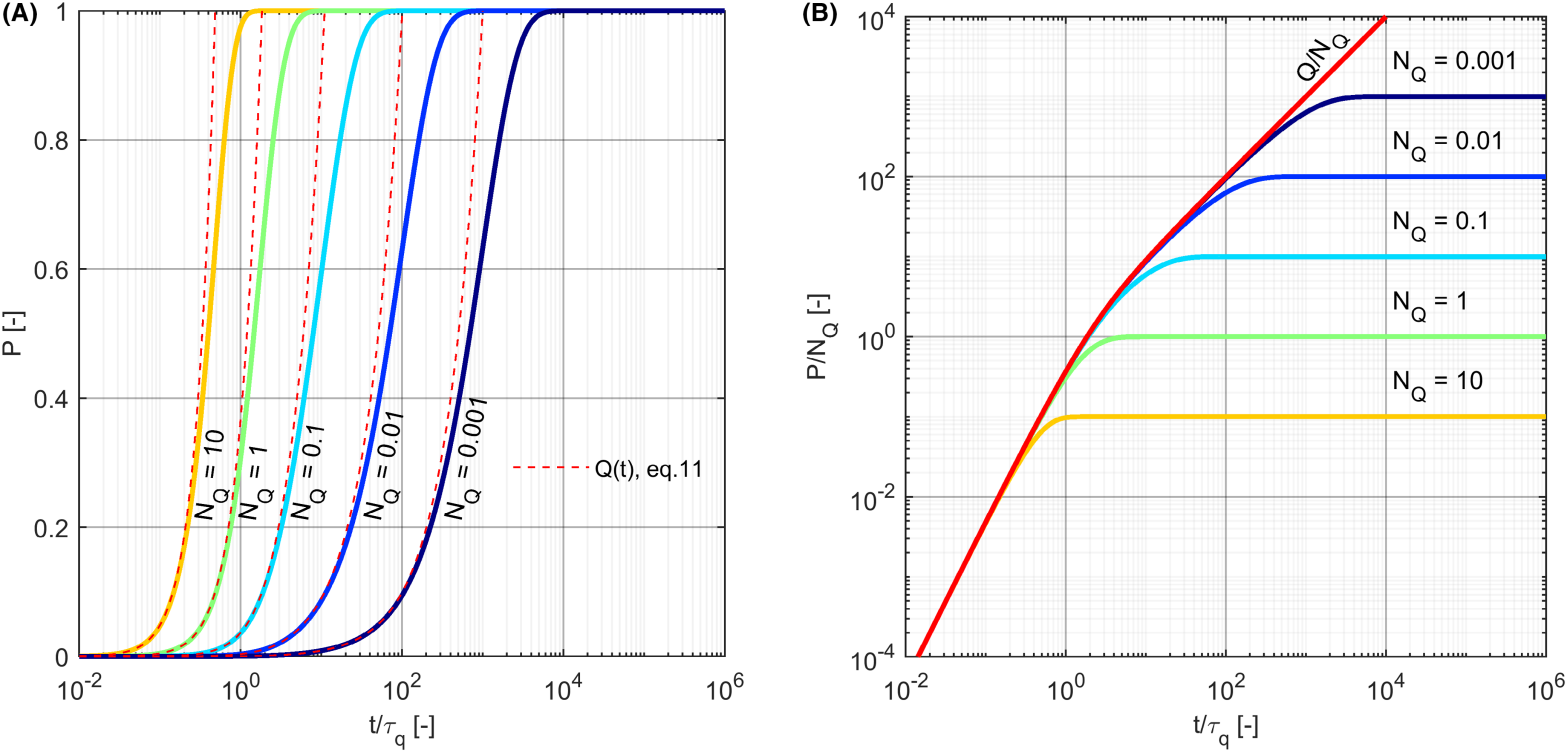
Left: The probability of infection as a function of time, $P_{\inf}\left(t\right)$, for different values of the normalized infectious dose $N_{Q}$: 10 (orange), 1 (green), $10^{-1}$ (cyan), $10^{-2}$ (blue), $10^{-3}$ (navy blue). The red dashed lines depicts the small‐probability approximation $Q\left(t\right)=N_{Q}\left(t/\tau_{q}+e^{-t/\tau_{q}}-1\right)$ (Equation 9). Right: The probability of infection normalized by $N_{Q}$ and compared with $Q/N_{Q}$ (red line)

The system of Equations 1 and 7 is the G‐N model,^27^ and can be solved to obtain the probability of infection. Inserting Equation 8 into Equation 3 and integrating, we obtain the amount of virus inhaled by a susceptible individual,

(9) $$Q\left(t\right)=N_{Q}\left(t/\tau_{q}+e^{-t/\tau_{q}}-1\right),$$

where the dimensionless number,

(10) $$N_{Q}=\theta_{q}\frac{\tau_{q}^{2}}{\tau_{b}}=\left(\frac{\theta_{q}}{\tau_{q}^{-1}}\right)\left(\frac{\tau_{b}^{-1}}{\tau_{q}^{-1}}\right),$$

is the normalized infectious dose that has been inhaled, which combines the characteristic times (or rates) relevant to the process; and $\tau_{b}^{-1}=\frac{b}{V}$ is the breathing rate of the susceptible individual ($\tau_{b}$ [−] is the characteristic time of the pulmonary activity). Notice that all characteristic times in Equation 9 are rescaled by the virus removal time. $N_{Q}$ is the product of the normalized quanta emission rate and breathing rate; hence, the inhaled viral dose grows linearly with the breathing and quanta emission rates and decreases quadratically with the removal rate. Inserting Equation 9 into Equation 2, we obtain the probability of infection as a function of the dimensionless time $t/\tau_{q}$,

(11) $$P_{\inf}\left(t\right)=1-e^{-Q\left(t\right)}=1-e^{-N_{Q}\left(t/\tau_{q}+e^{-t/\tau_{q}}-1\right)},$$

which depends only on the normalized infectious dose $N_{Q}$ and increases more rapidly with time for larger values of $N_{Q}$ (Figure 1a).

As long as the risk is small, the probability of infection can be approximated by the infectious dose ($P_{\inf}\left(t\right)\approx Q\left(t\right)$) and exhibits three distinct regimes (see Figure 1b): (i) a quadratic regime at early time ($t/\tau_{q}\ll 1$) when the virus removal is negligible and the viral concentration of the indoor air grows linearly in time; (ii) a transition regime at $t/\tau_{q}\approx 1$; and (iii) a linear regime at later time ($t/\tau_{q}\gg 1$) when quanta emission and removal rates are at equilibrium and the viral concentration of the indoor air remains constant. The infectious dose provide an excellent approximation of the infection probability: it overestimates the correct value by only $5\cdot 10^{-5}$ for $P\left(t\right)=0.01$ (i.e., $Q\left(t\right)=0.01005$), by $5.3\cdot 10^{-3}$ for $P\left(t\right)=0.1$ (i.e., $Q\left(t\right)=0.1053$), and by $2.3\cdot 10^{-2}$ for $P\left(t\right)=0.2$ (i.e., $Q\left(t\right)=0.223$). When the probability of infection increases, it deviates from the infectious dose, which represents an upper bound for the infection risk. Notice that for large values of $N_{Q}$, the probability can deviate from $Q\left(t\right)$ before it enters the linear or the transition regime; whereas for small values of $N_{Q}$ the probability deviates from $Q\left(t\right)$ only after the quadratic regime has been reached.

## 3 THE PROBABILITY OF INFECTION WITH STOCHASTIC QUANTA EMISSION

The probability of infection that we have derived in the previous sections assumes that breathing, virus removal, and quanta emission rates are known. If we consider these parameters as stochastic variables in order to account for their variability and uncertainty, Equation 11 must be interpreted as the conditional probability of infection,

(12) $$P_{\inf}\left(t|N_{Q},\tau_{q}\right)=1-e^{-N_{Q}\left(t/\tau_{q}+e^{-t/\tau_{q}}-1\right)},$$

whereas the (marginal) probability of infection is the expected value of the conditional probability,

(13) $$P_{\inf}\left(t\right)=E_{N_{Q},\tau_{q}}\left[P_{\inf}\left(t|N_{Q},\tau_{q}\right)\right]=\iint P_{\inf}\left(t|N_{Q},\tau_{q}\right)\mathrm{dP}\left(N_{Q},\tau_{q}\right),$$

where $P\left(N_{Q},\tau_{q}\right)$ is the joint probability for $N_{Q}$ and $\tau_{q}$, which are, in general, not independent because the normalized infectious dose depends on the virus removal rate (see Equation 10).

### 3.1 A stochastic model of quanta emission

In the following, we will assume that the variability of the breathing and removal rates are small compared with the variability of the quanta emission rates, which on the contrary can vary over orders of magnitude across infectious individuals; hence, we will threat only the quanta emission rate, $\theta_{q}$, as stochastic variable. The quanta emission rate is given by the sum of the emission rates of all infectious individuals in the enclosed space,

(14) $$\theta_{q}=\sum_{i=1}^{I}\theta_{q,i},$$

where I is the number of infected individuals, and $\theta_{q,i}$ is the quanta emission rate of the i‐th individual. Therefore, we write the probability as

(15) $$P\left(\theta_{q}\right)=\sum_{I=1}^{N}P\left(\theta_{q}|I\right)B\left(I;N,\pi \right),$$

which is the product of the conditional probability $P\left(\theta_{q}|I\right)$ and the binomial distribution,

(16) $$B\left(I;N,\pi \right)={\left(1-\pi \right)}^{N-I}\pi^{I}\left(\begin{array}{c} N\\ I \end{array}\right)={\left(1-\pi \right)}^{N-I}\pi^{I}\frac{N!}{I!\left(N-I\right)!},$$

which describes the probability that I of the N occupants are infectious when the prevalence of the infection (i.e., the fraction of infectious individuals in the general population) is π.

If the individual emission rates follow a log‐normal distribution,^34^ we can use the Fenton–Wilkinson method to approximate their sum as a log‐normal random variable.^35^, ^36^ By assuming that the emission rates of all individuals in the enclosed space belong to the same log‐normal distribution (i.e., $\log\theta_{q,i}∼N\left(\mu ,\sigma^{2}\right)$), we have that

$$\log\theta_{q}∼N\left(\mu_{I},\sigma_{I}^{2}\right),$$

where the variance and the mean are $\sigma_{I}^{2}=\log\left[\frac{e^{\sigma^{2}}-1}{I}+1\right]$ and $\mu_{I}=\mu +\log I+\frac{\sigma^{2}}{2}-\frac{\sigma_{I}^{2}}{2},$ respectively.

Recalling that $N_{Q}=N_{Q}\left(\theta_{q},\tau_{q},\tau_{b}\right)$ and $P\left[N_{Q}\left(\theta_{q},\tau_{q},\tau_{b}\right)\right]=P\left(\theta_{q}\right)$, we can write Equation 13 as

(17) $$P_{\inf}\left(t\right)=\sum_{I=1}^{N-1}E_{\theta_{q}|I}\left[P_{\inf}\left(t|N_{Q},\tau_{q}\right)|I\right]B\left(I;N,\pi \right),$$

where

(18) $$E_{\theta_{q}|I}\left[P_{\inf}\left(t|N_{Q},\tau_{q}\right)|I\right]=\int P_{\inf}\left(t|N_{Q},\tau_{q}\right)\mathrm{dP}\left(\theta_{q}|I\right).$$

The conditional probability of infection, $P_{\inf}\left(t|N_{Q},\tau_{q}\right)$, is given by Equation 12, whereas the conditional probability of quanta emission, $P\left(\theta_{q}|I\right)$, follows a log‐normal distribution, and $B\left(I;N,\pi \right)$ is the binomial distribution that describes the probability that I of the N individual in the enclosed space are infectious when the prevalence is π and varies according to the epidemiological situation (the case I=N is not included in the sum because at least one occupants must be susceptible for an infection to occur).

Notice that Equation 17 assumes that all non‐infectious individuals are susceptible (hence, there is no immunity resulting from vaccination or recovery from a previous infection). To account for preexisting immunity, in Equation 17, we should introduce the binomial probability that only S of the N−I non‐infectious individuals are susceptible, that is, the infection probability would be $\sum_{I=1}^{N-1}\sum_{S=1}^{N-I}E_{\theta_{q}|I}\left[P_{\inf}\left(t|N_{Q},\tau_{q}\right)|I\right]B\left(I;N,\pi \right)B\left(S;N-I,\pi \right)$. The infection probability in Equation 17 expresses the expected fraction of the N−I susceptible individuals that are infected during their stay in the enclosed space, whereas the individual risk of infection for a specific susceptible individual is

(19) $$R_{\inf}\left(t\right)=\sum_{I=1}^{N-1}E_{\theta_{q}|I}\left[P_{\inf}\left(t|N_{Q},\tau_{q}\right)|I\right]B\left(I;N-1,\pi \right).$$

Notice that the individual risk is not affected by preexisting immunity because it is conditional to the fact that the individual under consideration is susceptible.

### 3.2 Quanta emission rate of SARS‐CoV‐2

In case of respiratory diseases, the emission of infectious viral copies into the environment occurs through virus‐laden droplets and aerosol that are produced by infectious individuals during respiratory events such as sneezing, coughing, breathing, or talking (see, e.g., Refs [37, 38]). As we are interested in indoor airborne transmission, we focus on breathing and talking, which can represent a sustained source of viral copies during the prolonged stay of an infectious individual indoors. The emitted respiratory droplets and aerosol display a large size variability that affects their behavior, with large droplets rapidly depositing to the ground, whereas small and intermediate droplets quickly evaporate into aerosol that remains suspended in the air for hours.^1^, ^6^, ^39^ The droplets size may exhibit a multimodal distribution (see, e.g., Refs [39, 40]), but log‐normal distribution is in general used to describe experimental data, particularly to describe the droplets of small and intermediate size.^33^, ^34^, ^39^, ^41^

The quanta emission rate of an infectious individual, $\theta_{q,i}$, depends on the number and size distribution of respiratory droplets and aerosol emitted over time, as well as on the amount of viral copies carried by each of them. Models translating the emission rate of respiratory droplets and aerosol by an infectious individual into a viral emission rate are very rare. Here, we use the model proposed by Buonanno, Morawska, and Stabile^42^; Buonanno, Stabile, and Morawska^34^ to express the emission rate of SARS‐CoV‐2 in quanta of viral copies per hour, and recently extended to describe also other respiratory diseases.^43^ Relying on the droplet size distribution measured by Morawska et al.,^44^ on the SARS‐CoV‐2 concentration found in the sputum, and on the copies‐to‐quanta conversion factor estimated by Watanabe et al.^45^ for SARS‐CoV‐1, they performed Monte‐Carlo simulations to derive the probability density functions of the quanta emission rate of an infectious individual exercising different activities. To account for the strong variability between individuals (e.g., in the measured viral copies concentration of the sputum), they assume a unimodal log‐normal distribution with mean and standard deviation given in Table 1 (data from Ref. [46]). While the variance is fixed, the mean of $\log\theta_{q,i}$ strongly depends on the activity of the infectious person and increases with the metabolic rate and when talking. Despite the approximate nature of the model and the large uncertainty remaining on its parameters, the model proposed by Buonanno, Morawska, and Stabile;^42^ Buonanno, Stabile, and Morawska^34^ allows us to quantify the emission of airborne viral copies in terms of quanta of infection and accounts for the variability of the input parameters.

**TABLE 1:** Mean, μ, and standard deviation, σ, of the logarithm of the quanta emission rate of an infectious individual, $\log\theta_{q,i}$, according to the model of Buonanno, Stabile, and Morawska^34^ (data from Ref. [46] but presented using the natural logarithm); also shown are the mean, ${\bar{\theta }}_{q,i}$, of $\theta_{q,i}$ [1/h], and the breathing rate $\tau_{b}^{-1}$ [m${}^{3}$/h]

Activity	μ	σ	${\bar{\theta }}_{q,i}$ [h${}^{-1}$]	$\tau_{b}^{-1}$ [m${}^{3}$/h].
Resting	−0.990	1.66	1.47	0.49
Standing	−0.852	1.66	1.69	0.54
Light activity	0.046	1.66	4.14	1.38
Heavy activity	0.921	1.66	9.93	3.30
Standing and speaking	0.759	1.66	8.45	0.54
Standing and speaking loud	2.487	1.66	47.5	0.54

*Note*: Mean, μ, and standard deviation, σ, of $\log\theta_{q,i}$; ${\bar{\theta }}_{q,i}$, mean of $\theta_{q}$ [h${}^{-1}$]; breathing rate, $\tau_{b}^{-1}$ [m${}^{3}$/h].

## 4 MEASUREMENT CAMPAIGNS IN CABLE CARS

The viral concentration in the air and the probability of infection are strongly dictated by the ventilation rate, E, which is able to remove infectious viral copies from the air more efficiently than gravity deposition or inactivation. In case of natural ventilation, ventilation rate is generally not known with a sufficient level of precision. In cable cars, ventilation is provided by the natural airflow through the openings that is driven by the aerodynamic load generated by the apparent wind speed (i.e., the vector sum of the cable‐car traveling velocity and the external wind velocity). The interaction between the apparent wind and the shape of the gondola can lead to a complex three‐dimensional flow separation, which is sensitive to the external conditions and makes it difficult to estimate E based on simplified assumptions.

**FIGURE 2:**
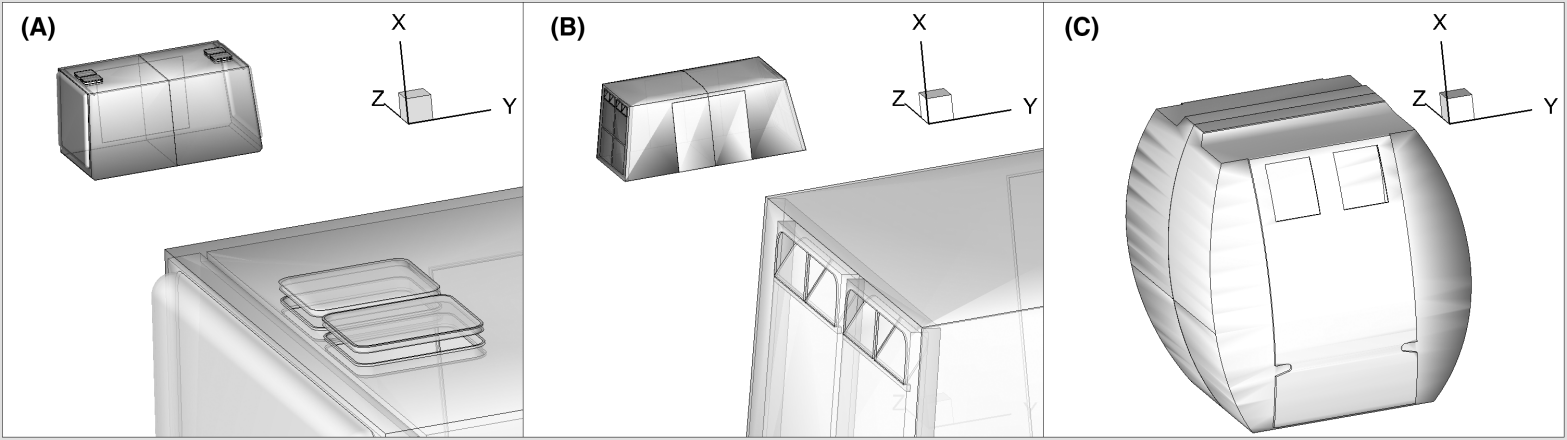
Three‐dimensional views of the three cable car types: (A) ROOF (77‐LPB, CWA Constructions AG) with rooftop ventilation; (B) FLAPS (80‐LPB, CWA Constructions AG) with leading and trailing wall ventilation flaps; and (C) OMEGA3 (8‐KBK CWA Constructions AG) with side windows. The cable car traveling direction lies in the X−Y plane

To overcome this difficulty, we measured the airflow velocity and estimate the ventilation rates in three different types of cable cars installed in the Engelberg district (Obwalden, Switzerland) and characterized by different openings, passenger capacity, volume, and traveling time. The most relevant geometrical parameters, the passenger capacity, and the main operational parameters are summarized in Table 2. The first cabin (ROOF, 77‐LPB CWA, Constructions AG) is equipped with roof openings, the second one has openings on the windward and leeward walls (FLAPS, 80‐LPB, CWA Constructions AG), whereas the third one has two windows on one of the side walls (OMEGA3, 8‐KBK CWA Constructions AG) (see Figure 2). These three cabin types are representative of most common configurations that can be found in rope‐way installations.

**TABLE 2:** Main geometric and operational parameters for the three cable‐cars in this study

Cable‐car	V	W	H	L	Capacity	Nom. Speed	Travel time	Elevation gain	Ground distance
	$\left[m^{3}\right]$	$\left[m\right]$	$\left[m\right]$	$\left[m\right]$	$\left[N\right]$	$\left[m/s\right]$	$\left[\min\right]$	$\left[m\right]$	$\left[m\right]$
ROOFTOP	49	3.27	2.7	5.47	77	9.5	5	531	2190
FLAPS	39	3.07	2.4	5.86	80	9	4.5	627	1975
OMEGA3	5.3	n.a.	n.a.	n.a.	8	6	9 + 7	781 + 644	2650 + 1996

*Note*: The cable cars are installed in the Engelberg district, Switzerland. The cable car OMEGA3 has also an intermediate station.

**FIGURE 3:**
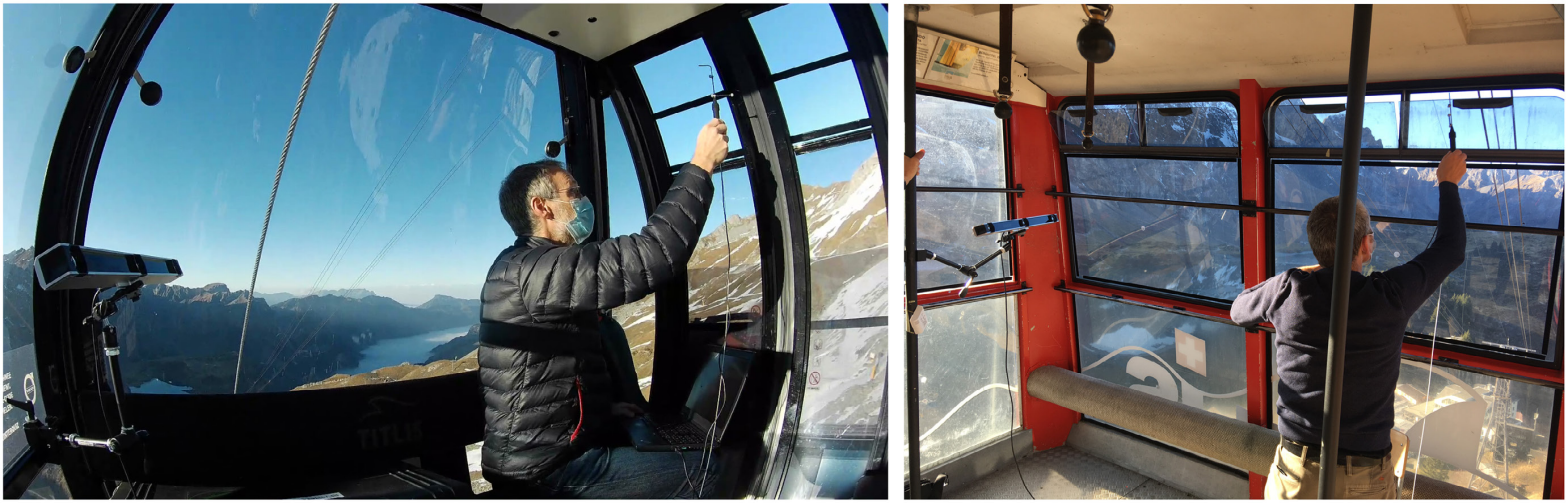
Airflow velocimetry measurements in the OMEGA3 gondola (left) and ROOF cable car (right). In each picture, the three integrated cameras of the 3D tracking system are visible on the left‐hand side, whereas the operators hold the sensor stick equipped with fiducial marks for precise positioning of the acquired data

The ventilation rate E is calculated from three‐dimensional (3D) volumetric velocimetry data measured close to the cable‐car ventilation openings. Two different sensors have been employed to sample the velocity field in distinct field measurements: a pneumatic 7‐hole probe (Surrey Sensors Ltd.) and a compact 3D ultrasonic anemometer (Trisonica Mini, Anemoment), which is able to measure velocity as low as 0.1 m/s. The probes are integrated in a 3D tracking camera system (Procap, Streamwise Gmbh) that records the position of the 3D probe with a tracking frequency of 120 Hz and a position error smaller than 1 mm (Figure 3). The system allows on‐line monitoring of the flow data and automatically corrects the measured velocity to account for the relative motion of the probe due to unwanted vibrations or position drifts. The point‐wise sampled values are used to reconstruct the 3D time‐averaged flow field on a 30×30×30 mm Cartesian grid. Geometrical calibration by means of fiducial marks allows the air velocity data to be accurately positioned on the 3D CAD model of the geometry.

**FIGURE 4:**
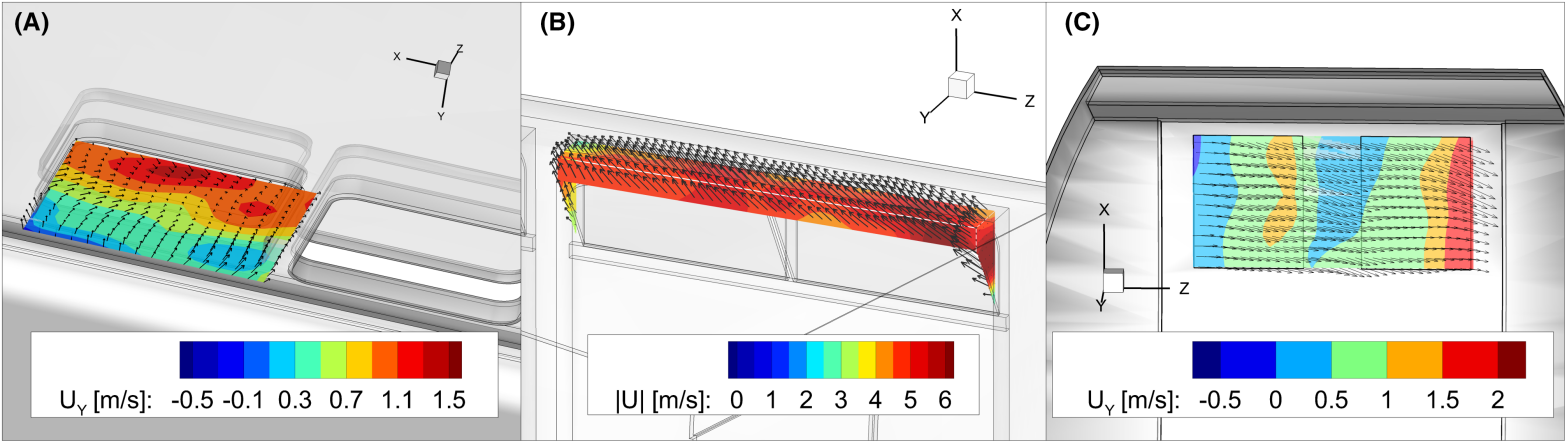
3D vector plots and contour maps of: (A) velocity component $U_{Y}$ along the Y axis normal on a XZ plane close to the openings of the cable car ROOF, (B) absolute velocity U on XZ and XY planes close to the openings in cable car FLAPS, and (C) velocity component $U_{Y}$ along the Y axis normal on a XZ plane close to the openings of the cable car OMEGA3

The field measurements were conducted on November 11, 2020, and on May 6, 2021. During the first day of measurement the weather conditions were stable and the external wind speed was almost negligible. On the contrary, the second day of measurement was characterized by considerable wind gusts with peak speed up to 16 m/s; a snowfall also added additional challenges as the values recorded by the sensor may be affected by the presence of snowflakes in the measurement volume. In order to reduce the time averaging error, we sampled the flow field during different runs (typically 2–4). For each cable car, we extracted the velocity from the measured data at selected planes (as shown in Figure 4) and integrated the normal component of the velocity to estimate the incoming flux. The uncertainty on the ventilation rate calculated from the incoming flux was estimated from the residual of the divergence of the velocity in the measurement volume. As the divergence of the velocity field is expected to be zero, its residual provides us with a confidence interval for the estimated velocity field and, by error propagation, on the ventilation rate. The estimated flow rates and air exchange times are reported in Table 3 together with the weather conditions recorded at the closest weather station (Engelberg Titlis, SLF/WSL).

**TABLE 3:** Measured air flow rates, exchange time and weather conditions during the measurement recorded by closest weather station (long. 8${}^{\circ }$25′/lat. 46${}^{\circ }$47′ altitude 2149 m, source: Eidg. Institut für Schnee‐ und Lawinenforschung, WSL)

Cablecar type	Probe	E	$\tau_{e}$	$\tau_{e}^{-1}$ (AER)	T	Average wind speed	Peak wind speed
‐	‐	$\left[m^{3}/s\right]$	$\left[s\right]$	ACPH	[${}^{\circ }C$]	$\left[m/s\right]$	$\left[m/s\right]$
ROOF	7‐hole	0.60±0.20	80±30	45±15	1.6	1.3	3
FLAPS	7‐hole	1.51±0.02	25.8±0.3	140±2	4.8	1.3	3
FLAPS	7‐hole	1.92±0.04	20.3±0.4	177±4	1.05	5.4	16
FLAPS	3D‐sonic	1.41±0.16	28±3	130±15	1.05	5.4	16
OMEGA3	7‐hole	0.26±0.02	20.6±1.6	177±14	4.0	1.3	3
OMEGA3	7‐hole	0.18±0.05	29±8	120±30	0.3	2.6	10
OMEGA3	3D‐sonic	0.09±0.06	60±40	60±40	0.3	2.6	10

*Note*: The confidence intervals of E, $\tau_{e}$, and $\tau_{e}^{-1}$ are calculated by error propagation of the confidence interval of the velocity field in the measurements volume, which is estimated from the residual of the divergence of the estimated velocity field.

Abbrevations: ROOF, cable car of type 77‐LPB CWA Constructions AG; FLAPS cable car of type 80‐LPB, CWA Constructions AG; OMEGA3 cable car of type 8‐KBK CWA Constructions AG; E, ventilation rate; $\tau_{e}$, air exchange time; AER, air exchange rate; ACPH, air changes per hour [h${}^{-1}$].

The cable car with rooftop ventilation (ROOF) exhibits complex airflow patterns that are well illustrated by the inwards velocity contour plot depicted in Figure 4a, which displays both positive (inward) and negative (outward) velocity values at the same opening. As the ventilation openings could find themselves in a separation region originated by the external flow, this configuration may be very sensitive to the ambient parameters and to the cable‐car traveling direction. In case of lateral windows (OMEGA3), the collected data displays high variability with an estimated air exchange rate that varies by up to a factor of three (from 60 to about 180 air changes per hour). This variability is due to the different external wind conditions observed during the three measurements. The instability of the external wind conditions (peak gust speed and direction) can modify the flow around the cable car and generate flow separation regions that may entrain the side windows, hence reducing the effectiveness of the ventilation. In contrast, the data collected in the cable car of the FLAPS type were rather consistent despite the very different wind conditions (Table 2). This is due to the position of the ventilation openings that are located on the wall facing the traveling direction: in this configuration, the component of the apparent wind velocity has higher magnitude (up to 5 m/s) in the direction normal to the opening plane and no separation region can be generated at the windward wall.

In general, the variability observed in more challenging situations (May 6, 2020) is part of cable car operation in high mountain environment and can reduce the effectiveness of natural ventilation; on the contrary, the measurements performed in calm winds (November 11, 2020) are more reproducible and stable. Also, based on the estimated measurement uncertainty, we assume the following air exchange rates as representative of the three cable car types: 45 air changes per hour (ACPH) for ROOF; 160 ACPH for FLAPS; and 120 ACPH for OMEGA3.

## 5 PROBABILITY OF INFECTION IN CABLE CARS

With the quanta emission rate model of Buonanno, Morawska, and Stabile^42^; Buonanno, Stabile, and Morawska^34^ (Section 3.2) and the air exchange rates estimated from the measurements presented in Section 4, we compute the probability of infection during a cable car journey. From a SARS‐CoV‐2 half‐life of 1.1 h^47^ and a deposition rate of 0.24 h${}^{-1}$ ^34^, ^48^ we assume a constant inactivation rate $\lambda =0.87\;h^{-1}$, which will be used in the following calculations. Notice that the contribution of the inactivation rate (and of the deposition rate, in particular) to the virus removal rate is important if the ventilation is poor or absent, but it becomes negligible for the air exchange rates observed in cable cars.

**FIGURE 5:**
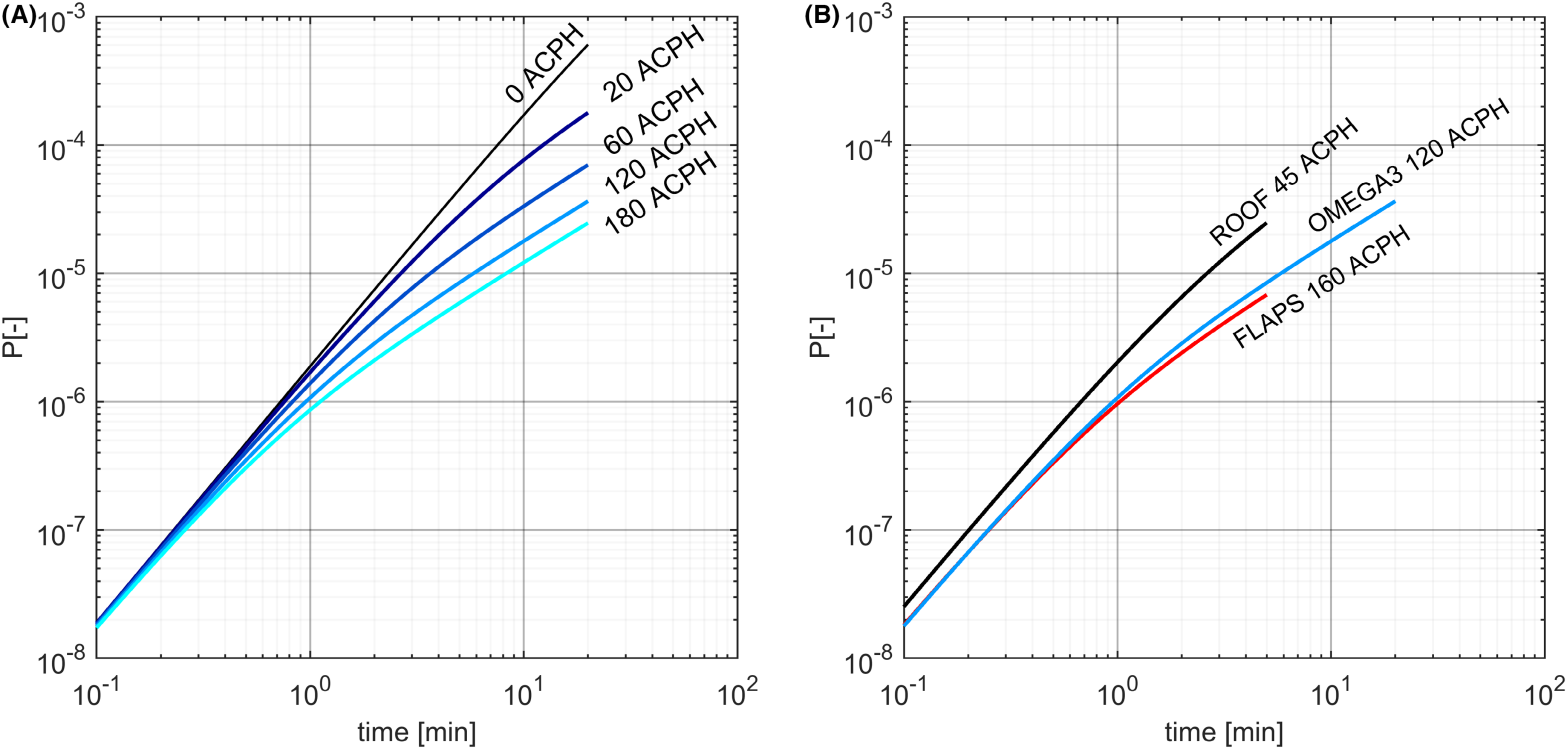
Left (A). The probability of infection in cable cars of OMEGA3 for different air exchange rate: 180 ACPH (cyan), 120 ACPH (light blue), 60 ACPH (blue), 20 ACPH (navy) and no air exchange (black). Right (B). Probability of infection as a function of time in the three types of cable car: ROOF (black, 45 ACPH), FLAPS (red, 160 ACPH), and OMEGA3 (blue, 120 ACPH). It is assumed that the prevalence is 1%, that the cable cars travel at full capacity (i.e., 77, 80, and 8 passengers for ROOF, FLAPS, and OMEGA3, respectively), and that the breathing rate and the potential quanta emission rate of the passengers are comparable to a standing person (see Table 1)

The ventilation rate plays a crucial role in controlling the probability of infection. Assuming a prevalence of 1%, in Figure 5, we plot the probability of infection at full passenger capacity for the typical traveling times of the different cable cars (around 5 min for ROOF and FLAPS, and up to 20 min for OMEGA3). In the OMEGA3 with open windows, the estimated virus removal time, $\tau_{q}$, varies between 20 and 60 s and the probability of infection grows linearly after a couple of minutes, reaching $2.2\cdot 10^{-5}$ at the end of the journey for 180 ACPH, and increasing to $3.6\cdot 10^{-5}$ and $7\cdot 10^{-5}$ for 120 and 60 ACPH, respectively (Figure 5a). In absence of ventilation, the removal time is determined only by deposition and virus inactivation, $\tau_{q}=\lambda$, and the probability of infection grows quadratically and reaches $6\cdot 10^{-4}$ at the end of the traveling time. As the cable cars are never completely airtight, this case provides an upper bound for the OMEGA3 with closed windows and indicates that the infection probability drops by at least an order of magnitude when the windows are open. As the three types of cable cars have a similar passenger density (between 1.5 and 2 passengers per cubic meter), they have a similar infection probability in the early quadratic regimes. (Notice that, for all plots presented hereafter, the infection probability in case of no ventilation can always be extrapolated by prolonging the quadratic regime, as long as the virus inactivation rate can be neglected—see also the discussion in Section 2.3) However, when the ventilation rate becomes important, ROOF shows a higher infection probability at given time than FLAPS and OMEGA3 (Figure 5b). As a result of the longer travel time, a journey in OMEGA3 has a slightly higher infection probability than a journey in FLAPS ($6.8\cdot 10^{-6}$) and ROOF ($2.5\cdot 10^{-5}$).

**FIGURE 6:**
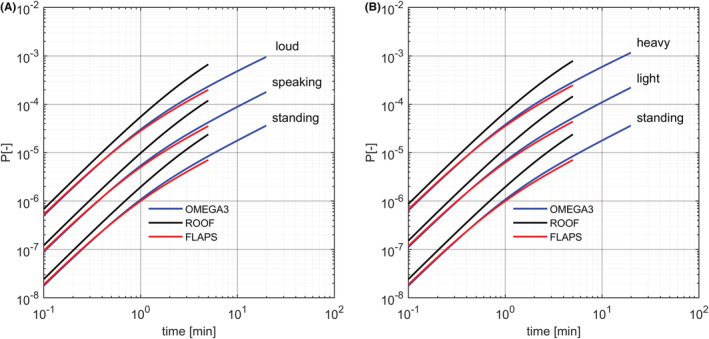
Effects of vocalization and different metabolic activities on the probability of infection in the three cable cars traveling at full passengers capacity for typical air exchange rates (black, ROOF with 77 passengers and 45 ACPH; red, FLAPS with 80 passengers and 160 ACPH; and blue, OMEGA3 with 8 passengers and 120 ACPH). We assume a prevalence of 1%. Breathing rate and the potential quanta emission rates of the passengers are taken form Table 1. Left (A): increase in infection probability with the voice volume of the passengers; from the lower to the higher curves we assume that all passengers are standing silently, standing and speaking, and standing and speaking loud. Right (B): increase in infection probability with the metabolic activities of the passengers; from the lower to the higher curves we assume that all passengers are standing, lightly exercising, and heavy exercising

In general, there is a large uncertainty on the quanta emission model due to both a lack of sufficient quantitative knowledge on the emission of infectious viral copies and on the virological parameters, and to differences in the activities of the passengers. In Figure 6, we compare the three cable cars when the vocalization and the metabolic activity of the passenger increase. We observe an increase in infection probabilities of about 25 times with the vocalization and about 25–35 times with the metabolic activity.

In all cases that we have considered, the probability of infection remains small and the inhaled infectious dose provides an excellent approximation (i.e., $Q\left(t\right)-P_{\inf}<5\cdot 10^{-7}$ for $P_{\inf}\left(t\right)<0.001$). Hence, we can write Equation 17 as

(20) $$P_{\inf}\left(t\right)\approx {\bar{N}}_{Q}\left(t/\tau_{q}+e^{-t/\tau_{q}}-1\right),$$

where

(21) $${\bar{N}}_{Q}=\sum_{I=1}^{N-1}E_{\theta_{q}|I}\left[N_{Q}|I\right]P\left(I;N,\pi \right)=e^{\mu +\sigma^{2}/2}\frac{\tau_{q}^{2}}{\tau_{b}}\sum_{I=1}^{N-1}\mathrm{IP}\left(I;N,\pi \right)\approx \pi \frac{N}{V}\left(b{\bar{\theta }}_{q,i}\right)\tau_{q}^{2},$$

and is the expectation of the normalized infectious dose, and

(22) $${\bar{\theta }}_{q,i}=e^{\mu +\sigma^{2}/2}$$

is the mean value of the individual quanta emission rate, $\theta_{q,i}$, which is log‐normally distributed according to the Fenton–Wilkinson approximation. Notice that the last approximation requires that π is sufficiently small, or N sufficiently large, such that $N\cdot P\left(N;N,\pi \right)=N\pi^{N}$ can be neglected with respect to the sum, $\sum_{I=1}^{N-1}\mathrm{IP}\left(I;N,\pi \right)$. Equations 20 and 21 provide great insights into the dependency of the infection probability on the cable‐car parameters. As long as the probability remains small (we recall from Section 2.3 that Equation 20 overestimates the exact value only by $5.3\cdot 10^{-3}$ for $P_{\inf}=0.1$ and by $2.3\cdot 10^{-2}$ for $P_{\inf}$ as high as 0.2), the infection probability increases linearly with the prevalence, π, with the passenger number density, N/V, and with ${\bar{\theta }}_{q,i}$, which depends on the metabolic activity and on the vocalization, and expresses a characteristic quanta concentration in the exhaled air. In the initial quadratic regime ($t/\tau_{q}\ll 1$), the infection probability is independent of the viral removal time,

(23) $$P_{\inf}\left(t\right)\approx \pi \frac{N}{V}\frac{\left(b{\bar{\theta }}_{q,i}\right)}{2}\;t^{2},$$

whereas in the linear regime ($t/\tau_{q}\gg 1$) it is inversely proportional to the removal rate, which can be approximated by the air exchange rate, $\tau_{e}^{-1}$, if the latter dominates the other viral removal mechanisms; hence we have

(24) $$P_{\inf}\left(t\right)\approx \pi \frac{N}{V}\frac{\left(b{\bar{\theta }}_{q,i}\right)}{\tau_{e}^{-1}}\;\left(t-\tau_{e}\right).$$

## 6 COMPARISON WITH OTHER INDOOR SETTINGS

The analysis presented in the previous sections allows us to estimate the infection probability in all indoor settings in which the virus transmission is mainly airborne. For small to moderate values, the time‐dependent infection probability is fully determined by the expectation of the normalized inhaled dose, Equation 21, which can be calculated once the occupant number density and the air exchange rate are known. The main source of uncertainty are the epidemiological and virological parameters, that is, the prevalence and, most of all, the quanta emission rate, which remains poorly characterized and difficult to estimate experimentally. Therefore, rather than discuss specific infection risk values for SARS‐CoV‐2, it is more instructive to compare the infection probability estimated for cable cars with those calculated for other enclosed spaces.

We first consider two other means of transportation: a train transporting 38 passengers in a 45 m^3^ car, and an aircraft carrying 200 passengers in a 400 m^3^ cabin. Typical train ventilation systems are expected to operate at air exchange rates up to 15 ACPH, with at most 8 APCH from external fresh air supply. Notice that these are rather an upper bound for normal operation conditions and markedly smaller rates were measured and reported in the literature (e.g., a total of about 5 ACPH, less than 2 ACPH of which are from outdoor air, by Ref. [49]). We can envisage two situations: in the most optimistic scenario, the ventilation system comprises HEPA filters that are assumed to have close to 100% efficiency in blocking virus‐laden aerosol and the total of 15 ACPH contributes to the viral removal rate; instead, if the filter has negligible efficiency in blocking virus‐laden aerosol, only 8 ACPH contributes to virus removal. In the case of an aircraft cabin, certification regulation requires a minimum of 5 L/s of fresh air per person at standard cabin conditions, a value that can be increased to around 8 L/s in new aircrafts for improved comfort.^50^, ^51^ Aircraft‐cabin ventilation is organized by compartments, with preferential flow across seats in the same row. Notice, however, that this does not prevent the diffusion of particles across a few adjacent seat rows^51^, ^52^ and, in any case, the passenger number density, which is the decisive parameter for infection probability, is not affected by compartmentalized ventilation.

**FIGURE 7:**
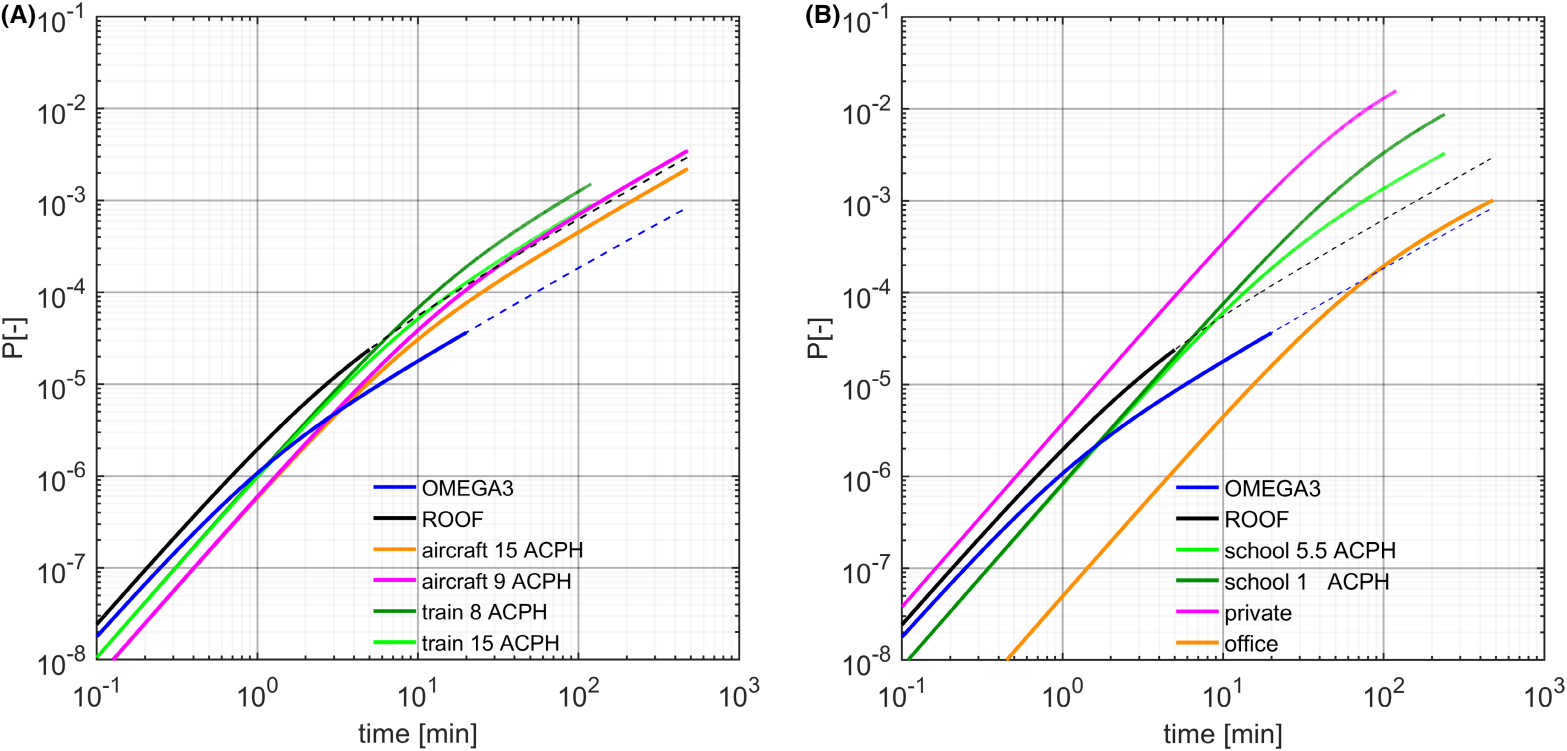
Comparison between the infection probability in cable cars and in other enclosed space. We plot the infection probability in the ROOF (black) and the OMEGA3 (blue) cable cars. (For illustration we extend the solution well beyond the typical journey time of 5 and 20 min for the ROOF and the OMEGA3 – dashed lines). We assume a prevalence of 1% and, if not stated otherwise, quanta emission and breathing rate correspond to a standing person (this is used as a proxy of persons with low metabolic activity but not resting). Left (A) Comparison with other means of transportation: 200 passengers in a 400 m^3^ aircraft cabin with 15 ACPH (orange) and 9 ACPH (magenta); 38 passengers in 45 m^3^ train car with 15 ACPH (light green) and 8 ACPH (dark green). Right (B), comparison with building space: two people working 8 h in 20 m^2^ × 2.5 m at 1.2 ACPH (orange); 20 pupils talking in a classroom of for 4 h 50 m^2^ × 3 m at 5.5 ACPH (light green) and 1 ACPH (dark green); 8 people gathering and talking loud for 2 h in a 30 m^2^ × 2.5 m private living room with no ventilation (magenta)

After the first couple of minutes, a ventilated cable car (OMEGA3) traveling with open windows shows noticeably lower infection probability than trains and aircraft (Figure 7a). This results from a much higher air exchange rate (up to 15–20 times larger) at comparable passenger density (between 0.5 and 1.5 passengers per m${}^{3}$). If we consider the journey duration, the probability of infection in a one‐hour (resp. two‐hour) train ride is at least 20–30 times (resp. 50–80 times) larger than in a typical 10 min ride in a ventilated OMEGA3 or in a typical 5 min ride in a ROOF. In aircrafts and trains the effects of the different ventilation rates becomes relevant after about 5 min, when we enter the transition regime, and the probability of infection varies proportionally to the inverse of the air exchange rate at later times (Equation 24).

Building spaces are also prone to accumulation of potentially virus‐laden aerosol, which can lead to high risk of infection. We consider three situations: two persons continuously working for 8 h in a 20 m^2^ × 2.5 m office; 20 pupils talking in a 50 m^2^ × 3 m classroom; and eight people talking loud during a dinner event in a private living room of 30 m^2^ × 2.5 m. We assume that the two public‐space scenarios comply with the ASHRAE standard for ventilation,^53^ which correspond to 1.2 and 5.5 ACPH in the office and in the classroom, respectively. In contrast, we assume no ventilation during the private social gathering (windows are kept closed). After a few minutes, the infection probability in the classroom and in the private gathering is markedly higher than in the cable cars (Figure 7b). This is due to the potentially higher quanta emission rate due to vocalization and to the much lower air exchange rate. A single cable car ride of typical duration has an infection probability lower by two to almost three orders of magnitude than a two‐hour social gathering or half a day spent in a classroom; and about 50 times lower than 8 h spent in an office for two.

Let us again assume a prevalence of 1% and consider a susceptible person commuting by train an hour a day (half an hour per journey) and working 8 h in an office together with a colleague. We can use Equation 19 to calculate the risk of infection in the public transport ($R_{\inf}\left(0.5h\right)=3.13\cdot 10^{-4}$) and in the office ($R_{\inf}\left(8h\right)=5.14\cdot 10^{-4}$); composing these estimates we obtain a total risk of infection of $1.14\cdot 10^{-3}$. Estimating 6 rides per ski day in cable cars and assuming an average individual risk for a susceptible person of about $2\cdot 10^{-5}$ per ride, we obtain a total individual risk of $1.2\cdot 10^{-4}$, hence an order of magnitude smaller than in the example of the commuter. For comparison, the individual risk of a susceptible pupil spending 4 h in a classroom is $3.1\cdot 10^{-3}$, whereas for a person attending a private gathering for 2 h in a non‐ventilated living room we have $1.37\cdot 10^{-2}$, that is more than two orders of magnitude higher.

## 7 CONCLUSIONS

The field campaigns to measure the airflow velocity in cable cars have allowed us to estimate the air exchange rates, which is a rather elusive parameters in naturally ventilated enclosed spaces and can critically depend on ambient parameters and weather conditions. We have estimated from 45 up to 180 ACPH depending on the operating conditions, on the cable‐car characteristics, and on the position of the ventilation openings. In general, cable cars with openings on the windward wall exhibit minimal dependence of the air exchange rate on ambient conditions (it has been consistently estimated in the range between 130 and 180 ACPH) and should be preferred when the objective is to achieve the highest ventilation rate. In popular gondola lifts equipped with side windows (like the OMEGA3), we have also estimated up 180 ACPH, but the ventilation rate may be affected by the external wind conditions, which may give rise to separation regions in the vicinity of the windows (this phenomenon is expected to occur also in cable cars equipped with roof openings, such as ROOF, in which we measured 45 ACPH). All experimental measurements performed in cable cars traveling with open windows have recorded much higher air exchange rates than in means of transportation equipped with mechanical ventilation (such as train cars and aircraft cabins) or in common indoor spaces such as offices and classrooms, for which current regulations prescribe much lower ventilation rates.

The knowledge about the ventilation rate allows us to compute the probability of infection and the individual risk of a susceptible individual potentially exposed to SARS‐CoV‐2. Here, we have developed a general stochastic infection model that is applicable to arbitrary enclosed spaces. We have neglected the droplet route of transmission which is likely to play a secondary role in indoor infections and could be effectively controlled by social distancing (if space allows) or using personal protective equipments such as masks or respirators. Instead, we have focused on the airborne transmission, which occurs through viral aerosols that remain suspended in the air for hours, accumulating indoors and leading to high risk of infection. We have assumed that the indoor air is well mixed, which is a justified assumption for viral small droplets and aerosols with long settling time and in presence of high air exchange rates that enhance mixing. In our model, we consider the viral emission rate as a stochastic variable, which depends on the probability that infectious individuals are present; therefore, the probability of infection is a function of the prevalence and the epidemiological situation.

As a results of the high air exchange rate and the comparatively shorter stay, the infection risks in cable cars traveling with open windows is remarkably lower than in other means of transportations and in building spaces. Accounting for the typical duration of the stay, the probability of infection during a cable‐car journey is lower by two to three orders of magnitude than in the other examples considered (the highest risk being estimated in case of a private gathering in a poorly ventilated room where people are talking loud). We estimate that the risk of infection of a commuter during a typical day (two half‐an‐hour journeys by train and 8 h spent in the office with a colleague) is one order of magnitude higher than a typical ski day with six rides in cable cars.

In most realistic situations, the expectation of the normalized inhaled dose (Equation 20) is an excellent approximation (and always an upper bound) for the infection probability. If the duration of the stay is longer than the typical air exchange time, the potential viral concentration of the indoor air is dictated by the equilibrium between the viral emission by the infectious occupants and the viral removal by ventilation (Equation 24). For practical purposes, the infection probability can be approximated by a simple linear formula,

(25) $$P_{\inf}\left(\Delta t_{s}\right)\approx \frac{\rho_{N}\Delta t_{s}}{\tau_{e}^{-1}}\cdot \left[\pi \left(b{\bar{\theta }}_{q,i}\right)\right],$$

where $\Delta t_{s}$ is the duration of the stay, and $\rho_{N}=N/V$ the occupant number density, which can be readily computed from the number of occupants, N, and the volume of the enclosed space, V. The air exchange rate, $\tau_{e}^{-1}$, can be estimated, for instance, by standard dilution measurements that employ carbon dioxide as tracer. A rough estimate can also be obtained by simply comparing the carbon‐dioxide concentration recorded during two rides with the same occupants, one with closed and the other with open windows. Once the rate of CO${}_{2}$ production by the occupants is obtained from the time derivative of the CO${}_{2}$ concentration recorded with closed windows (assuming no air exchange), the ventilation rate is estimated by comparison with the time evolution of the CO${}_{2}$ concentration recorded during the ride with open windows. The estimate is particularly simple if the equilibrium between production and removal of carbon dioxide is reached during the journey with open windows.

In Equation 25, the quantity in brackets depends on the epidemiological parameters (the prevalence, π) as well as on the product of the typical breathing rate, b, and the typical viral emission rate of an infectious individual, ${\bar{\theta }}_{q,i}$. These parameters are the most uncertain, because the prevalence is only approximately known during an epidemic, and because reliable viral emission rate models are difficult to obtain, particularly for new viruses such as SARS‐CoV‐2. Nevertheless, Equation 25 allows us to reliably compare the probability of infection between two indoor situations also if the emission model is inaccurate or even if it is not available. For instance, assuming the same activity level and emission model, for a given prevalence the relative risk with respect to a reference indoor situation can be simply calculated from the duration of the stay, the occupant number density, and the air exchange rate.

Finally, we remark that the stochastic infection model is general and can be applied to all indoor situations in which the viral transmission is predominately airborne and the indoor air is well mixed. Under these conditions, our approach and findings are applicable to any enclosed space and allow us to assess the effectiveness of simple mitigation measures such as increased ventilation or reduction of the maximum occupancy in shared indoor spaces.

## CONFLICT OF INTEREST

We declare no conflict of interest.

## Data Availability

The data that support the findings of this study are available from the corresponding author upon reasonable request.
